# The biodisposition and hypertrichotic effects of bimatoprost in mouse skin

**DOI:** 10.1111/exd.12071

**Published:** 2013-01-02

**Authors:** David F Woodward, Elaine S-H Tang, Mayssa Attar, Jenny W Wang

**Affiliations:** 1 Department of Biological Sciences, Allergan, Inc.Irvine, CA, USA; 2 Department of Pharmacokinetics and Disposition, Allergan, Inc.Irvine, CA, USA

**Keywords:** bimatoprost, hair, metabolism, prostanoid, skin

## Abstract

Studies on bimatoprost were performed with two objectives: (i) to determine whether bimatoprost possesses hair growth-stimulating properties beyond eyelash hypertrichosis and (ii) to investigate the biodisposition of bimatoprost in skin for the first time. Bimatoprost, at the dose used clinically for eyelash growth (0.03%) and given once daily for 14 days, increased pelage hair growth in C57/black 6 mice. This occurred as a much earlier onset of new hair growth in shaved mice and the time taken to achieve complete hair regrowth, according to photographic documentation and visual assessment. Bimatoprost biodisposition in the skin was determined at three concentrations: 0.01%, 0.03% and 0.06%. Dose-dependent *C*_max_ values were obtained (3.41, 6.74, 12.3 μg/g tissue), and cutaneous bimatoprost was well maintained for 24 h following a single dose. Bimatoprost was recovered from the skin only as the intact molecule, with no detectable levels of metabolites. Thus, bimatoprost produces hypertrichosis as the intact molecule.

## Background

Bimatoprost was originally designed as an ocular hypotensive and has been extensively used for treating glaucoma 1,2. Eyelash hypertrichosis was observed as a side effect, and bimatoprost effects on eyelash growth have now been studied in detail 3–5. Because bimatoprost effects on other hair types have not been reported, we conducted studies on mouse pelage skin 6,7. The cutaneous biodisposition of bimatoprost was also investigated because the presence of substantially intact bimatoprost would be indicative of prostamide receptor involvement 8–13.

## Questions addressed

1. Do the hypertrichotic properties of bimatoprost extend beyond eyelashes, for example mouse pelage hair 6,7?
2. Is bimatoprost metabolically converted in skin? Substantial levels of intact bimatoprost would indicate prostamide receptor mediation 8–13.

## Experimental design

Pelage hair growth was studied in C57/black 6 mice. The animals were shaved, and hair growth was evaluated by photographic documentation and visual assessment as 1 the time for onset of hair regrowth and 2 the time taken to completely cover the shaved area with new hair. Bimatoprost, at the dose used clinically to treat eyelash hypotrichosis, was given once daily for 14 days. The duration of the experiment was 42 days.

Bimatoprost studies on skin biodisposition were conducted at 0.01%, 0.03% and 0.06% doses. Blood samples were also collected for analysis. Two biodisposition studies were performed, one for 24-h duration and the other for 21-day duration.

## Results

**Figure 1:**
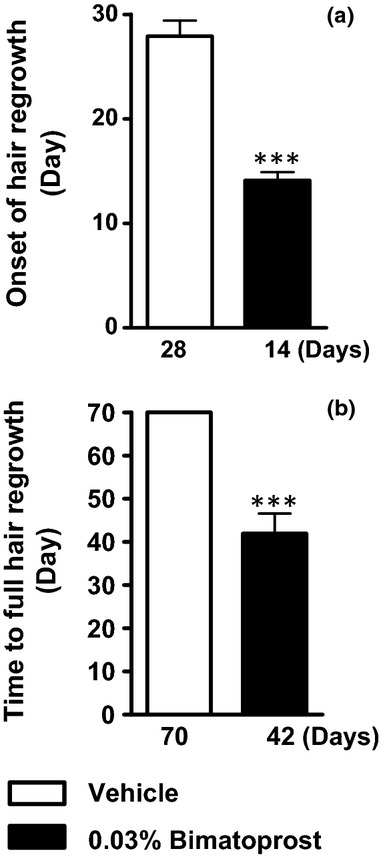
The effect of topically applied 0.03% bimatoprost on (a) the onset of hair regrowth and (b) the time for full hair regrowth to cover the shaved area. Open histogram blocks represent animals that received vehicle; filled histogram blocks represent animals that received 0.03% bimatoprost once daily for 14 days. Values are mean ± SEM, *n* = 10, ***P < 0.001 according to Student's non-paired *t*-test.

The effects of once-daily bimatoprost on pelage hair growth are summarised in Fig 1. The time of onset of hair regrowth was essentially halved (Fig. 1a). More importantly, the time taken to cover the shaved back with regrown hair was dramatically and highly significantly reduced (Fig. 1b). Interestingly, bimatoprost appeared to produce a uniform regrowth of hair over the shaved area, rather than radiating out from a central locus as was observed for the control group.

**Figure 2:**
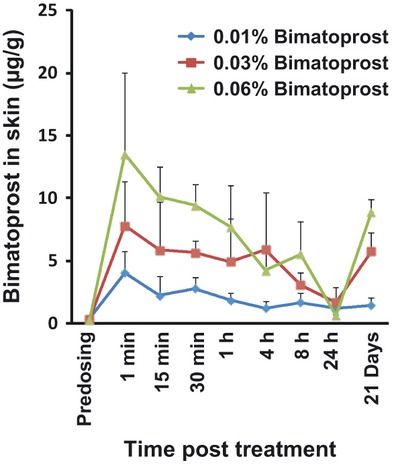
The biodisposition of graded doses of bimatoprost (0.01%-blue, 0.03%-red, 0.06%-green) in mouse skin. This graphical depiction combines the results from a single-dose, 1-day study and a once-daily, 21-day study where only the 21-day skin drug levels were determined. Points represent mean values ±SEM, *n* = 5.

The cutaneous drug levels of graded doses of bimatoprost are depicted in Fig. 2. High concentrations of bimatoprost were rapidly achieved in the skin, and these remained relatively well maintained for 24 hr at about 1 μg/g tissue. In a subchronic 21-day study, no substantial drug accumulation was apparent, but a clear dose–skin concentration relationship was apparent. Supplementary tables provide the *C*_max_ values, areas under curve and absolute concentrations for both blood and skin. Bimatoprost was found only as the intact molecule in both skin and blood, with no evidence of hydrolytic conversion to 17-phenyl PGF_2α_.

## Conclusions

These studies show, for the first time, the biodisposition of bimatoprost in skin together with its effects on hair growth. The effects of bimatoprost on mouse pelage hair growth were investigated at the same dose as that employed for treating eyelashes 3,4. Cutaneous levels of bimatoprost achieved were dose dependent and were well maintained over a 1-day period. Results from a 21-day study provided no evidence for bimatoprost accumulation on repeated dosing with a 0.01% dose, but some accumulation was apparent for the 0.03% and 0.06% doses. Bimatoprost remained as the intact molecule, indicating that it exerts its effects on hair growth by stimulating prostamide receptors 8–13.

Bimatoprost essentially remained as the intact molecule in mouse skin; the putative enzymatic hydrolysis product (17-phenyl PGF_2α_) was only detected twice in a total of 270 separate analyses of different skin and blood samples. Previously, in mouse eyes, it was shown that bimatoprost remains intact 14. Similarly, PGF_2α_-ethanolamide (prostamide F_2α_) remains without significant hydrolytic degradation in mouse blood 15. On comparing mouse skin and monkey ocular tissue bioavailability 8, it appears that bimatoprost accesses cutaneous tissue more readily and the tissue levels are better maintained than in ocular tissues. These data suggest that once-daily administration to the skin should be adequate to obtain optimal hair growth. This contention presumes that there is a relatively homogeneous distribution of bimatoprost between the hair follicle and the skin layers. A further consideration is that bimatoprost was applied once daily for only 14 days in the hair growth experiment, as an expedient based on limited and overextended manpower resources. It follows that the dosing regimen used in these present studies may have underestimated the effect of bimatoprost on hair growth.

Bimatoprost has long been established as potently effective as the intact molecule, with a pharmacological profile distinct from prostanoid FP receptor agonists and their ester prodrugs 8,9,12,16. The pharmacology of bimatoprost closely resembles that of prostamide F_2α_ 9–11. Further pharmacological characterisation has been achieved by designing selective prostamide antagonists 17–19 and structural elucidation of the prostamide receptor 13. The results herein indicate hair growth as a further prostamide-mediated effect that may be mimicked by bimatoprost.

Bimatoprost was almost invariably found in blood samples from mice that received topical bimatoprost on shaved skin. Blood levels were about one-thousandth of those present in skin. Although bimatoprost was detected in pharmacologically active levels in mouse blood, this would be greatly ‘diluted’ in the blood by humans as they are about 5000 times heavier/larger. The human scalp area of coverage would be about 10–50 times greater than that of the shaved mouse skin. Presuming similar penetration characteristics for bimatoprost in mouse and human skin, the likely blood concentration in humans would be in the range of 50 pg/ml. Anticipated human blood levels of 10–100 pg/ml are beneath the pharmacologically active levels for bimatoprost 20.

In summary, bimatoprost stimulates the growth of mouse pelage hair. Bimatoprost was found as the intact molecule in mouse skin and blood, indicating that it stimulates hair growth by interacting with prostamide-sensitive receptors 8–13. These studies indicate that the ability of bimatoprost to stimulate hair growth may extend beyond eyelashes, but effects on human scalp hair growth, for example, cannot necessarily be predicted from mouse pelage hair experiments. The human hair follicle expresses the prostanoid FP receptor 21, but expression of altFP4 13 is required to predict a positive outcome with bimatoprost.

## Supporting Information

**Data S1.** Materials and Methods.

**Table S1.** (a) Comparison of Bimatoprost pharmacokinetics in mouse skin and blood following 1 and 21 days of once daily dosing. (b) Pharmacokinetics of Bimatoprost biodisposition in mouse skin following single graded doses.
