# Supplementary material for: The biodisposition and hypertrichotic effects of bimatoprost in mouse skin
Source: Exp Dermatol. 2013 Jan 2;22(2):145–8. doi: 10.1111/exd.12071 (PMC3594970; doi:10.1111/exd.12071)
Supplement: Supplementary file 1 [file exd0022-0145-SD1.docx]

**Materials and Methods**

**Reagents**

Bimatoprost ( 17-phenyl trinor PGF2α-ethylamide ) was purchased from Torcan (Ontario, Canada), 17-phenyl PGF_2α_ from Cayman Chemical (Ann Arbor, MI, USA), deuterated standards from Organic Consultants (Eugene, OR, USA). Graded doses of bimatoprost were formulated in a vehicle of the following composition (% v/v) : glycerin 2%, propylene glycol 10%, diethylene glycol monoethylether 10%, ethanol 30%, carbomer 0.15%, triethanolamine 0.16%, purified water q.s. 100% ( pH 7.0 ).

**Bimatoprost Induced Hair Growth**

C57/Black/6 mice, 7-week old female mice (n=10), with free access to drinking water and food, were employed in piliation studies. The procedures have been approved by Allergan's Animal Care and Use Committee (AACUC). The dorsal hair of each animal was shaved with clippers (area ~2cm x 4cm). Dosing with Bimatoprost was once per day (QD) by topical administration . The drug was dosed daily for 14 days. Experimenters were unaware of the formulation identity . Photographic and visual observations were conducted for 42-days to determine the onset of new hair growth and the day of hair growth completion. In a preliminary study it was shown that mouse hair takes around 70 days to regrow and re-cover the shaved area (data shown in Figure 1b).

**Bimatoprost Pharmacokinetics and Drug Metabolism**

C57/Black6 mice were allowed free access to drinking water and food (UAR A04-purchased from VAR, Villemoisson s/Orge, France). The procedures have been approved by Allergan's Animal Care and Use Committee (AACUC). At least 5 days of acclimatization were allowed before commencing the experiment. Twenty four hours before application of Bimatoprost formulations, the backs of the animals were shaved using clippers and under general anesthesia. Further depilation was carried out using commercially available VEET®. Bimatoprost solutions were applied to the dorsal skin as a 0.1mL volume per day. The animals were euthanized at pre-determined times post-Bimatoprost dosing. Five animals were allocated to each time point. Back skin biopsies and blood samples were obtained in duplicate. Residual fat and connective tissue were gently removed from the underside of the skin samples by scraping . The skin specimens were then wiped with a gauze previously moistened by distilled water. To each skin biopsy sample 2.5mL of 50% acetonitrile: 50% methanol were added. Each 0.2mL blood sample was treated identically. Samples were stored at -20°C.

Bioanalysis: Sample preparation involved addition of internal standards = 10µL x 250ng/mL d_5_-Bimatoprost and d_4_-17-phenyl PGF_2α_. This was shaken for 5 min followed by centrifugation at 3000 rpm x 5 min. The supernantant was evaporated down to 0.3mL. This preconcentrate was diluted with 1.0mL of 2.5% formic acid before solid phase extraction using a C18 disc and an automated solid phase extractor (Zymark, Hopkinton, MA, USA). The extracts were evaporated to dryness and reconstituted in acetonitrile. The reconstituted samples were injected in a 100μL volume and analyzed by LC-MS/MS using a PE Sciex API 3000 mass spectrometer (Applied Biosystems, Foster City, CA, USA), with an auto sampler and HPLC pumps (Shimadzu Scientific Instruments, Columbia, MD, USA). Normal phase HPLC was performed using as APS-2 column 3μm, 2x150mm (Keystone, Lock Haven, PA, USA), with solvent gradient elution using acetonitrile/0.5% formic acid and methanol/0.5% formic acid mobile phase. Mass spectrometric detection involved positive-to-negative switch electrospray ionization and mass scanning in the multiple reaction monitoring (MRM) mode. The specific precursor-product ion pairs used in MRM analysis were as follows = m/z 398 (MH^+^-H_2_O) → 362 (Bimatoprost), m/z 403 → 367(d_5_ –Bimatoprost), m/z387 (M-H)^-^ →192 (17-phenyl PGF_2α_) and M/z 391-196 (d_4_-17-phenyl PGF_2α_). The analysis software (version 1.1, Applied Biosystems, Foster City, CA USA) integrated the peak area of analyte and internal standard and constructed calibration curves relating peak area ratios of analyte/internal standard to concentration of analyte.

**Results**

**Drug Metabolism**

Drug biodisposition studies were conducted at three doses 0.01%, 0.03%, and 0.06%. The dose employed for hair growth studies (0.03%) was thereby bracketed. Two studies were performed. A one day study where terminal blood and skin biopsy samples were obtained at times 0, 1, 15, and 30 min and 1, 4, 8, and 24hrs. A second study involved daily dosing for 21 days, in order to detect any cumulative events; analyses were performed at 1 and 21 day time points .The putative enzymatic hydrolysis product of Bimatoprost, 17-phenyl PGF_2α_, was analyzed at all time points in all biological samples. In a total of 270 samples, 17-phenyl PGF_2α_ was detected in only one blood sample (0.412ng/mL) and one skin sample (1.51 μg/g). Viewed in the context of the limit of detection (0.25ng/mL) and the amount of Bimatoprost detected (3.65ng/mL and 481 ng/g in these particular blood and skin samples respectively), 17-phenyl PGF_2α_ was a very minor species. The mean skin levels of Bimatoprost are depicted in Figure 2. Bimatoprost rapidly achieved an essentially maximal cutaneous concentration, which was essentially well-maintained over 24hrs (Figure 2). No evidence for accumulation was obtained when Bimatoprost was dosed once daily for 21 days at a 0.01% dose, while Bimatoprost produced dose-dependent increases in skin levels with some accumulation for the two higher doses at 21 days (Table 1a, supplemental ). Clear-cut difference in cutaneous bioavailability were obtained for the graded doses of Bimatoprost at early time points and the actual cutaneous concentrations achieved were high and well in excess of 1μg/g tissue . The C_max_ and area under curve values obtained for cutaneous Bimatoprost levels revealed an even clearer dose-response relationship (Table 1b, supplemental).

Blood levels of Bimatoprost associated with topical Bimatoprost applications to the skin were similarly analyzed. The appearance of Bimatoprost in blood showed a similar kinetic profile to the cutaneous results but, importantly, concentrations in blood (Tables 1a and 1b , supplemental ) were approximately one thousand times less than those found in skin. There was a modest spike immediately post-dosing.

|  | **Bimatoprost in skin (ng/g tissue)** | | **Bimatoprost in blood (ng/mL** | |
| --- | --- | --- | --- | --- |
|  | **DAY 1** | **DAY 21** | **DAY 1** | **DAY 21** |
| Bimatoprost 0.01% | 931 ± 245 | 721 ± 342 | - | 1.7 ± 1.1 |
| Bimatoprost 0.03% | 1330 ± 920 | 3610 ± 900 | 1.8 ± 0.8 | 1.5 ± 0.7 |
| Bimatoprost 0.06% | 550 ± 155 | 4930 ± 930 | 4.1 ± 2.9 | 5.9 ± 2.8 |

Table 1a. Comparison of Bimatoprost pharmacokinetics in mouse skin and blood following one day and twenty-one days of once daily dosing. Sampling points were 24hr and 21X 24hr. . Values are x̅ ± SEM; n = 5.

|  | **C_MAX_ (ng/g tissue)** |  | **AUC ng.hr/g tissue** |
| --- | --- | --- | --- |
| Bimatoprost 0.01% | 3410 ± 1089 |  | 32,100 ± 2718 |
| Bimatoprost 0.03% | 6740 ± 2616 |  | 74,600 ± 4821 |
| Bimatoprost 0.06% | 12,300 ± 2589 |  | 84,600 ± 4107 |

Table 1b. Pharmacokinetics of Bimatoprost biodisposition in mouse skin following single graded doses. C_max_ is maximal concentration achieved and AUC is area under curve over a 0-24 hr time interval. Values are x̅ ± SEM; n = 5.
